# High Glucose Level Promotes Migration Behavior of Breast Cancer Cells through Zinc and Its Transporters

**DOI:** 10.1371/journal.pone.0090136

**Published:** 2014-02-28

**Authors:** Tomoka Takatani-Nakase, Chihiro Matsui, Sachie Maeda, Satomi Kawahara, Koichi Takahashi

**Affiliations:** Department of Pharmaceutics, School of Pharmacy and Pharmaceutical Sciences, Mukogawa Women's University, Nishinomiya, Hyogo, Japan; Vanderbilt University, United States of America

## Abstract

**Background:**

The diabetes patients have been associated with an increased risk of mortality by breast cancer and there are difference between the breast cancer patients with diabetes, and their nondiabetic counterparts in the regimen choice and effects of breast cancer treatment. However, the pathophysiological relationships of diabetes and breast cancer have not yet been elucidated in detail. In this study, we investigate the breast cancer cell line, MCF-7 motility, which linked to invasion and metastasis, in high glucose level corresponding to hyperglycemia and the role of Zn and its transporter.

**Methodology/Principal findings:**

We demonstrated the significant motility of MCF-7 cultured in hyperglycemic level (25 mM glucose) in comparison to normal physiological glucose level (5.5 mM glucose). The other hand, the osmotic control medium, 5.5 mM glucose with 19.5 mM mannitol or fructose had no effect on migratory, suggesting that high glucose level promotes the migration of MCF-7. Moreover, the activity of intracellular Zn^2+^ uptake significantly increased in high glucose-treated cells in comparison to 5.5 mM glucose, and the mRNA expression of zinc transporters, ZIP6 and ZIP10, was upregulated in 25 mM glucose-treated cells. The deficiency of ZIP6 or ZIP10 and intracellular Zn^2+^ significantly inhibited the high migration activity in 25 mM glucose medium, indicating that Zn^2+^ transported via ZIP6 and ZIP10 play an essential role in the promotion of cell motility by high glucose stimulation.

**Conclusion/Significance:**

Zinc and its transporters, ZIP6 and ZIP10, are required for the motility stimulated with high glucose level. These findings provide the first evidence proposing the novel strategies for the diagnosis and therapy of breast cancer with hyperglycemia.

## Introduction

The statistical result analysis of diabetes and breast cancer has indicated that the diabetes has been associated with an increased risk of mortality by breast cancer [1], [2], [3]. Moreover, it has been reported that there are difference between the breast cancer patients with diabetes and their nondiabetic counterparts in the regimen choice and effects of breast cancer treatment [1], [2], [4]. These results suggest that the currently established therapy of breast cancer may not be suitable in the patients with diabetes and understanding the breast cancer in diabetes may provide avenues that lead to important clinical implications and methodologies. However, the pathophysiological relationships of diabetes and breast cancer have not yet been elucidated in detail.

Extracellular circumstances of cancer cells can influence growth and behavior of the cells, resulting in invasion, metastasis and tumor development. We demonstrated that breast cancer cell line, MCF-7 cultured with high glucose level corresponding to hyperglycemia, significantly promoted migratory activity compared with normal physiological glucose level, indicating the possibility that the hyperglycemia in diabetes mediates the motility of breast cancer cells. The development of new treatment strategy of breast cancer with high glucose level requires further detail analysis of the mechanisms, especially in the relation of the high glucose level and tumor migration.

Zinc (Zn), which is essential for many cellular processes, has also been reported to play a potential role for second messenger in signal pathway linked with various physiological actions [5], [6]. Moreover, the imbalances in Zn homeostasis cause disease states including Alzheimer's disease [7], diabetes [8], [9], [10], cancer [11] and others [5], [12]. In breast cancer patients, the level of Zn has been found to be lower in serum than healthy subjects and elevated in malignant tissues [13], [14], [15], [16], whereas in liver, prostate and gallbladder cancers, the level of Zn in the malignant tissues has been found to be decreased [13], [14], [17], [18], [19]. These recent reports suggest that Zn is implicated in breast cancer development. The intracellular Zn level is tightly controlled by several protein molecules called zinc-binding protein and zinc transporters [20]. These behaviors of Zn in and out of cell across membranes are maintained through two families including the ZRT IRT-like protein (ZIP) family that facilitates Zn influx into the cytosol, and zinc transporter (ZnT) family that facilitates Zn efflux from the cytosol. Some ZIP family members are reported to involve in aggressive cancer progression [11], [16], [21], [22], [23], [24], [25], [26], [27], [28]. ZIP6 is associated with histological grade of estrogen-receptor-positive breast cancer, and is positively related with the lymph node metastasis [16], [25], [26], [27]. ZIP6 also has been reported to regulate epithelial-mesenchymal transition (EMT) [21], [23], [24]. Moreover, ZIP10 has been reported to mediate the migration and invasive behaviors of breast cancer cells [28]. However, the role of Zn and the transporters in breast cancer cell migration stimulated with the high glucose level are still unknown, and the evaluation is strongly required to contribute to novel treatment strategies for breast cancer under hyperglycemia.

In this report, we demonstrate for the first time the relation between the motility of MCF-7 in high glucose level corresponding to hyperglycemia and the role of Zn and the transporters. Our findings show that high glucose level-induced cell migration was inhibited by ZIP6 or ZIP10 knockdown and by zinc chelation, suggesting the Zn transported via ZIP6 and ZIP10 has essential functions for the increased motility of MCF-7 in high glucose level.

## Materials and Methods

### Cell line and cell culture

Human breast cancer cell line MCF-7 was obtained from European Collection of Cell Cultures (ECACC; Salisbury, UK) and cultured in minimum essential medium (MEM, Invitrogen, Carlsbad, CA, USA) containing 5.5 mM D-glucose supplemented with 10% heat inactivated fetal bovine serum (FBS), 100 U/ml penicillin and 100 µg/ml streptomycin (Nacalai Tesque, Kyoto, Japan). For routine culture, cells were incubated in a standard humidified incubator at 37°C in 5% CO_2_.

### Scratch wound-healing Assay

MCF-7 cells were seeded in a 35 mm plastic dish at 5×10^5^ cells. After 24 h incubation, cells reached 100% confluency and a wound was produced at the center of the monolayer by scraping the cells with a sterile 10 µl pipette tip. After scratching, the dish was gently washed with PBS to remove the detached cells, and the cells were incubated in MEM containing 10% FBS with normal (5.5 mM), high (25 mM) D-glucose concentration, or an osmotic control (5.5 mM D-glucose plus 19.5 mM D-mannitol or D-fructose) for 24 h at 37°C in a humidified and equilibrated (5% v/v CO_2_) incubation. The wound area at 0 and 24 h after scratching was photographed using a microscopy system (Nikon, Tokyo, Japan), and the cell-free wound area was measured using NIH Image J software. The migration rate was determined by quantifying the area covered from the initial time to 24 h-points. Multiple views of each dish were examined, and three to seven independent experiments were performed.

### Observation of actin organization

MCF-7 cells were seeded at 1×10^4^ cells on 35 mm collagen IV-coated glass bottomed dish. The following day, the medium was replaced in MEM containing 10% FBS with normal (5.5 mM), high (25 mM) D-glucose concentration, or an osmotic control (5.5 mM D-glucose plus 19.5 mM D-mannitol). After 24 h, the cells were washed with PBS, fixed with 4% paraformaldehyde for 30 min and permeabilized with 0.2% Triton X-100 for 10 min. After blocking with 5% bovin serum albumin in PBS for 30 min, the cellular F-actin was stained with Alexa Fluor® 546 labeled phalloidin (Invitrogen) for 90 min. The cells were washed with PBS and analyzed using the Nikon fluorescent microscopy system (Tokyo, Japan).

### Cellular uptake of Zn^2+^

Analysis of Zn^2+^ uptake into cells was performed as previously described [29]. Cultured cells were incubated with 18 nM ZnCl_2_ in serum-free MEM that contained no detectable levels of Zn^2+^ for 60 min at 37°C in a humidified and equilibrated (5% v/v CO_2_) incubation. After washing with PBS, the cells were loaded with 5 µM zinc fluorescent sensor, Newport Green DCF (Invitrogen), for 30 min at 37°C in a humidified and equilibrated (5% v/v CO_2_) incubation. The cells were washed with PBS and the fluorescent intensities of the cells were analyzed using a FACSCalibur flow cytometer (BD Biosciences, CA, USA) at 488 nm laser excitation and a 515-545 nm emission filter.

### Semiquantitative expression analysis with reverse transcription-polymerase chain reaction (RT-PCR)

Total RNA was isolated from cultured cells and purified using the RNeasy Mini kit (Qiagen, Valencia, CA, USA) according to the manufacture's instructions. From each sample, equal amount of RNA (100 ng) was used in oligo dT primed cDNA synthesis reaction with the Superscript® III First-Strand Synthesis SuperMix (Invitrogen) following the manufacture's instructions. Equal volumes of cDNA (2 µl) from each reaction were used for PCR analysis using gene-specific primers to either human ZIP6 (forward: 5′-TCTGTCACAAATCCCCTTCA-3′, reverse: 5′-GGAGGGCTCTTGTGAGTCTG-3′), human ZIP10 (forward: 5′- CCTGGTTCCTGAAGATGAGG-3′, reverse: 5′-CATGGCAGAGAGGAGGTTGT-3′) or human GAPDH (forward: 5′-CAATGGAAATCCCATCACCATC-3′, reverse: 5′- TGAAGACGCCAGTGGACTCC-3′). The reactions were carried out at 95°C for 1 min, followed by 25 cycles of 95°C for 30 sec, 55°C for 30 sec, and 72°C for 1 min. PCR products were visualized after electrophoresis through a 2% agarose gel and stained by ethidium bromide (0.5 µg/ml).

### Construction of short-hairpin RNA expression vectors

shRNA-expressing pDNAs driven by human U6 promoter were constructed from pBAsi-hU6 Neo vector (Takara Bio Inc., Shiga, Japan) according to the manufacturer's instructions. Target sequences of human ZIP6 (GenBank accession no. NM_012319) and ZIP10 (GenBank accession no. NM_020342) were designed by Takara siRNA Design Support System (Takara Bio Inc.). The pBAsi-hU6 Neo vector, which transcribes a non-related sequence of RNA with partial duplex formation, was used as a control pDNA throughout the present study. Each pDNA was amplified in Escherichia coli top 10 (Invitrogen) and purified using a QIAGEN Endofree Plasmid Maxi Kit (QIAGEN GmbH, Hilden, Germany).

### shRNA-mediated gene attenuation

MCF-7 cells were cultured at a density of 5×10^5^ cells per 6 well plate. One day later, the cells were transfected with shRNA each for human ZIP6, ZIP10 and control sequences using Fugene 6 Transfection Reagent (Roche) following the manufacturer's instructions. The gene-silencing effect was analyzed 2 days after transfection by RT-PCR using gene-specific primers. The primers used for PCR were: human ZIP6, 5′-TCTGTCACAAATCCCCTTCA-3′ (forward), 5′-GGAGGGCTCTTGTGAGTCTG-3′ (reverse); human ZIP10, 5′-AGCACCACATGTTAAAAATAATGC-3′ (forward), 5′-CATGGCAGAGAGGAGGTTGT-3′ (reverse).

### Transwell migration assay

After 2 days of transfection with shRNA for ZIP6 or ZIP10, transwell migration assays were carried out using 8.0 µm pore size Costar Transwell inserts (Corning Inc., MA, USA). The cells were counted (2.5×10^4^ cells) and then added to the upper chambers of 24-well transwell plates and were cultured in MEM containing 10% FBS with normal (5.5 mM), high (25 mM) D-glucose concentration, or an osmotic control (5.5 mM D-glucose plus 19.5 mM D-mannitol). After 24 h, the motile cells at the bottom of the filter were fixed with 4% formaldehyde and stained with 4′, 6′-diamidino-2-phenylindole (DAPI, sigma). The number of migration cells was quantified by counting the stained cells under the Nikon fluorescent microscopy system (Tokyo, Japan).

### Statistical analysis

All statistical analyses were performed using GraphPad Prism (ver. 5.00; GraphPad, San Diego, CA, USA). One- or two-way analysis of variance (ANOVA) followed by Tukey's post hoc test or Bonferroni multiple comparison tests was used for multiple comparisons. Differences were considered significant when the calculated *p*-value was <0.05.

## Results

### Effect of high glucose level on the migration of MCF-7

**Figure 1:**
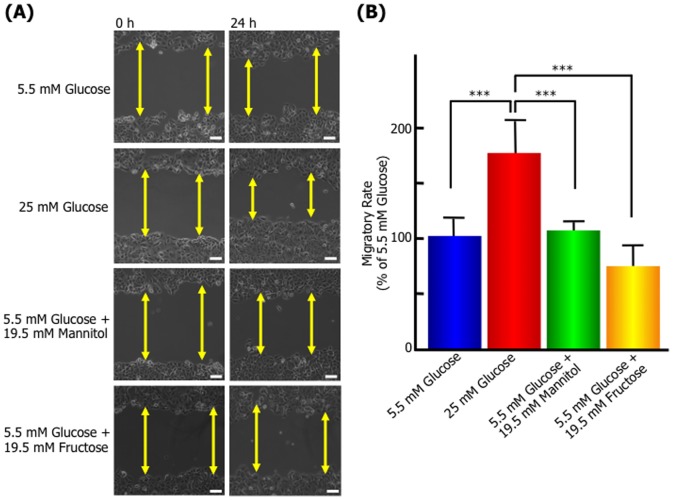
High glucose level promotes the migration of MCF-7 breast cancer cells. A confluent monolayer of MCF-7 cells was scratched and then cultured with normal (5.5 mM), high (25 mM) D-glucose concentration, or an osmotic control (5.5 mM D-glucose plus 19.5 mM D-mannitol or D-fructose) medium as described in the Materials and Methods. After 24 h, the wounded area was observed and photographed by microscopy. **(A)** Images at 0 and 24 h after scratching of each experimental condition. Arrowed lines represent cell-free zone in wounded area. Bars: 100 µm. **(B)** Migratory rates of each experimental condition. The quantity was normalized the wound closure area between 0 and 24 h against the wounded area at time zero and expressed as percentage of the migratory rate of MCF-7 cells incubated with the normal glucose concentration. Data are presented as means ± SD. ^***^ *p*<0.001 versus control (5.5 mM D-glucose concentration), n = 3–7.

**Figure 2:**
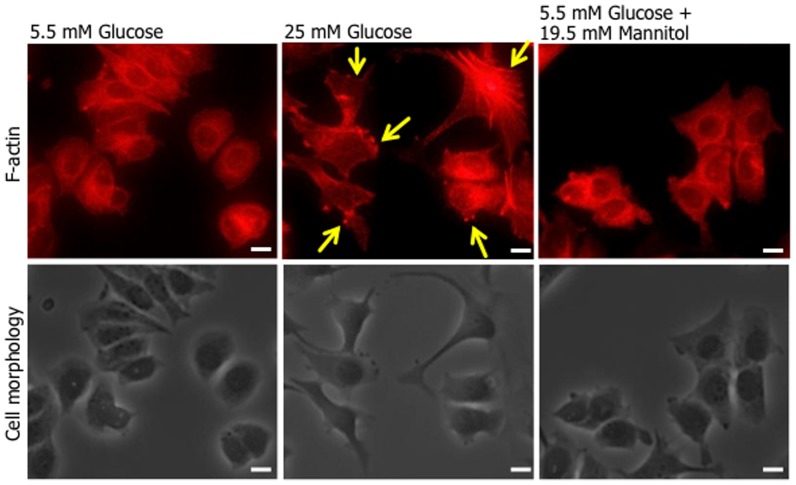
The formation of lamellipodia and morphology in MCF-7 cells cultured with high glucose level. MCF-7 cells plated on Collargen IV-coated coverslips were cultured with normal (5.5 mM), high (25 mM) D-glucose concentration, or an osmotic control (5.5 mM D-glucose plus 19.5 mM D-mannitol) medium for 24 h. Cells were fixed with 3.7% formaldehyde, permeabilised with 0.1% Triton X-100 and stained with Alexa Fluor®546-labelled phalloidin. Upper panels; fluorescence images, lower panels; phase-contrast images. Representative results of 3 independent experiments are shown. Arrows show representative lamellypodia formations. Bars: 10 µm.

To investigate the effect of high glucose level on MCF-7, we examined the cell migration in normal physiological glucose level (5.5 mM glucose) and hyperglycemic level (25 mM glucose) using the scratch wound-healing assay. Figure 1 shows representative photomicrographs and migratory rate of the result after 24 h of the scratch wound-healing assay. MCF-7 cultured with 25 mM glucose medium significantly promoted migratory compared with 5.5 mM glucose. However, the osmotic control medium, 5.5 mM glucose with 19.5 mM mannitol or fructose had no effect on migratory rate, suggesting that high glucose level promotes the migration of MCF-7. Moreover, 25 mM glucose medium also induces alternations in cell shape and actin organization, and significant lamellipodia formation was also observed for 25 mM glucose-treated cells (Figure 2). These observations suggest the induction of cell motility following high glucose stimulation.

### Zinc and ZIP are essential for glucose-induced cell migration

**Figure 3:**
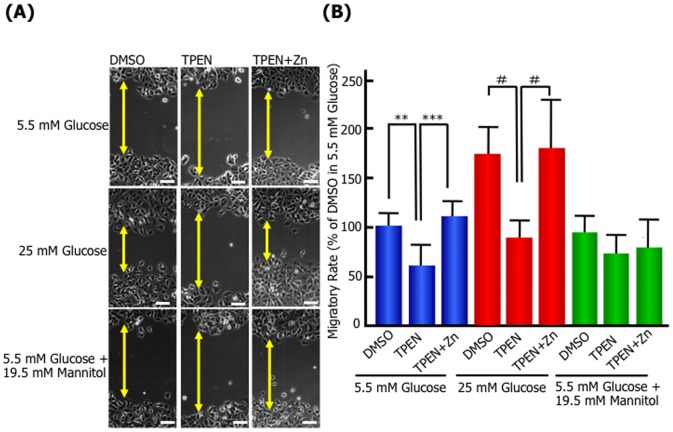
The effect of Zn^2+^-chelating reagent, TPEN on MCF-7 migration. A confluent monolayer of MCF-7 cells was scratched and then cultured in normal (5.5 mM), high (25 mM) D-glucose concentration, or an osmotic control (5.5 mM D-glucose plus 19.5 mM D-mannitol or D-fructose) medium in the absence or presence of 0.5 µM TPEN or 0.5 µM TPEN plus 5 µM ZnCl_2_. After 24 h, the wounded area was observed and photographed by microscopy. **(A)** Images at 24 h after scratching of each experimental condition. Arrowed lines represent cell-free zone in wounded area. Bars: 100 µm. **(B)** Migratory rates of each experimental condition. The quantity was normalized the wound closure area between 0 and 24 h against the wounded area at time zero and expressed as percentage of the migratory rate of MCF-7 cells incubated with the normal glucose concentration in the absence of TPEN. Data are presented as means ± SD. ^**^ *p*<0.01, ^***^ *p*<0.001, ^#^ *p*<0.0001, n = 7–18.

**Figure 4:**
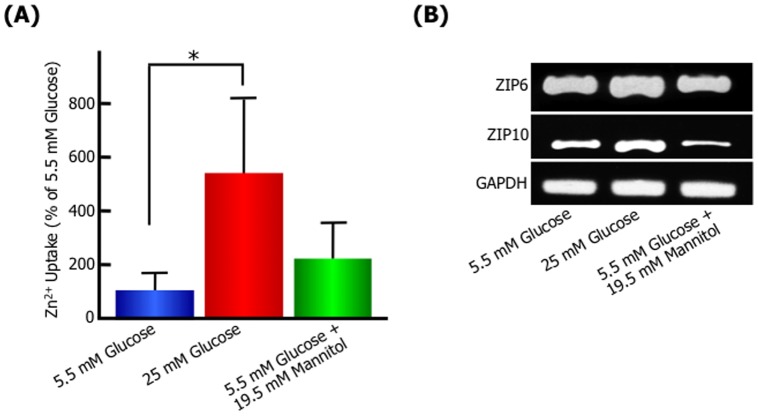
High glucose level induces intracellular Zn^2+^ uptakes and mRNA expression of ZIP in MCF-7. MCF-7 cells were cultured with normal (5.5 mM), high (25 mM) D-glucose concentration, or an osmotic control (5.5 mM D-glucose plus 19.5 mM D-mannitol) medium. After 24 h, uptake of Zn^2+^ in MCF-7 **(A)** and the changes in ZIP6 and ZIP10 mRNA levels in MCF-7 by RT-PCR **(B)** were analyzed. Data are presented as means ± SD. ^*^ *p*<0.05 versus control (5.5 mM glucose concentration), n = 4–5.

**Figure 5:**
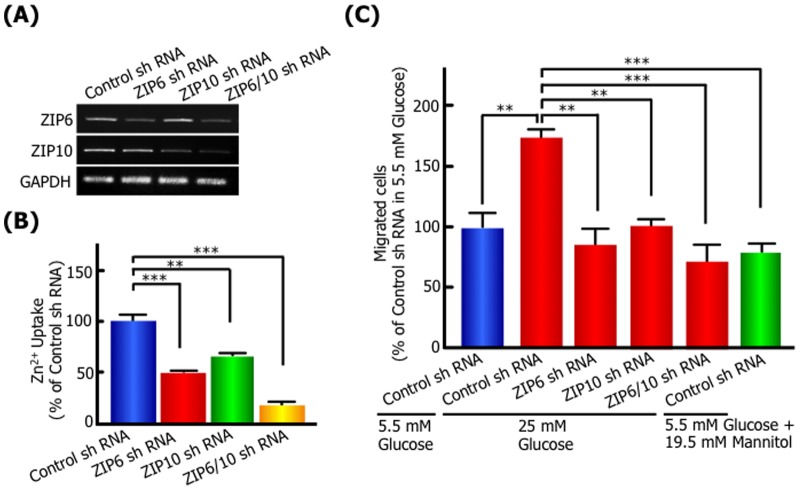
ZIP and intracellular zinc are essential for high glucose-induced migration. MCF-7 cells were transfected with shRNA each for ZIP6, ZIP10, and control sequences. The knockdown effect of ZIP6 shRNA and/or ZIP10 shRNA was shown in RT-PCR **(A)** and Zn^2+^ uptake activity in MCF-7 **(B)**. **(C)** In transwell migration assays, MCF-7 cells transfected each with shRNA for ZIP6 and ZIP10 were incubated with high (25 mM) D-glucose concentration for 24 h, and migrated cells were analyzed as described in Materials and Methods. Data are presented as means ± SD. ^**^ *p*<0.01, ^***^ *p*<0.001, n = 3.

Next, we have investigated whether the kinetics of Zn^2+^ are involved in high glucose-induced migration, because it has been reported that the expression level of ZIP6 or 10 is associated with the progression and metastasis of breast cancer [17], [20], [21], [22]. In the scratch wound-healing assay, the membrane-permeable zinc chelator, *N,N,N′,N′*-tetrakis (2-pyridylmethyl) ethylenediamine (TPEN) inhibited the migration of 25 mM glucose-treated cells (Figure 3). The inhibition was neutralized by the addition of free Zn^2+^ in the culture medium, suggesting intracellular zinc has essential functions in the high glucose-induced cell migratory activity. Moreover, the activity of intracellular Zn^2+^ uptake significantly increased in high glucose-treated cells compared with 5.5 mM glucose or 5.5 mM glucose with 19.5 mM mannitol (Figure 4A). The expression of ZIP zinc transporter, ZIP6 and ZIP10 mRNA was upregulated in 25 mM glucose-treated cells (Figure 4B). The osmotic control medium, 5.5 mM glucose with 19.5 mM mannitol had no stimulatory effect on Zn^2+^ uptake and ZIP6 and ZIP10 mRNA level. To further understand the role of ZIP6 or ZIP10 in the high glucose-induced cell migratory activity, we generated ZIP6-, ZIP10- or ZIP6 and ZIP10-knockdown MCF-7 cells using shRNA for human ZIP6, human ZIP10 and the control, and investigated the expression of ZIP, activity of Zn^2+^ uptake and migration. RT-PCR analysis of MCF-7 knockdown cells showed that mRNA expression of ZIP6 and ZIP10 was specifically depleted by each shRNA treatment (Figure 5A). The Zn^2+^ cellular uptake ability was significantly decreased in MCF-7 ZIP6, ZIP10, and ZIP6 and ZIP10-knockdown cells by 49.7%, 66.4% and 16.4%, respectively (Figure 5B). In a transwell migration assay as shown Figure 5C, the control cells showed high migration activity in 25 mM glucose medium by about 1.7-fold compared with 5.5 mM glucose or 19.5 mM mannitol, indicating similar results with wound healing assay. The high migration activity in 25 mM glucose medium was blocked by ZIP6, ZIP10, or ZIP6 and ZIP10 knockdown. These results suggested that Zn^2+^ transported via ZIP6 and ZIP10 play an essential role in the promotion of cell motility following high glucose stimulation.

## Discussion

In the present study, we demonstrated that the promotion of cell migratory ability was strongly dependent on glucose concentration and the Zn^2+^ transported via ZIP6 and ZIP10, which have essential functions for glucose-induced cell migration. The motility of breast cancer cells differ in normal physiological glucose level and hyperglycemic level, so these results highlight the need to consider the cell kinetics changed by extracellular circumstances for therapy of breast cancer with hyperglycemia.

Interestingly, the estrogen-receptor-negative breast cancer cell, MDA-MB-231 did not show enhanced migratory ability in high glucose level as results of MCF-7 (data not shown). The breast cancer with hyperglycemia is though to be much estrogen-receptor-positive breast cancer, because the patients with breast cancer and diabetes have been reported to be more likely to receive hormonal therapy than their nondiabetic counterparts [1], [4]. Therefore, there is a possibility of involvement of estrogen receptor and its pathways in the migratory stimulated high glucose level. In addition, Hela cells (cervical cancer) and DU-145 cells (prostatic cancer) also did not show enhanced migratory ability in high glucose level (data not shown), suggesting specificity of estrogen-receptor positive breast cancer cell for sensitivity in hyper glycemia as clinical statistical data. Recently, the mechanism of enhanced cancer cell motility has been reported to get involved with the EMT [30], [31], [32]. However, the reduction of E-cadherin as EMT marker in MCF-7 cultured with high glucose level for 24 h was not shown (data not shown), and our data indicated that the dynamic alternations of cytoskeleton and the up-regulation of intracellular Zn^2+^ are strongly associated with glucose-induced cell migration. Zinc has been shown to be a novel intracellular second messenger [5], [6], and it is possible that zinc plays an important role for the signal pathway of the cell motility in high glucose stimulation, although further studies are required. Intracellular Zn^2+^ concentrations are regulated by zinc transporters [20], and we demonstrated that ZIP6 and ZIP10 play a crucial role in the uptake of Zn^2+^ and migratory promotion in high glucose stimulation. It has been suggested that the expression of ZIP6 mRNA is positively correlated with the lymph node metastasis in estrogen-receptor-positive breast cancer [27], therefore, ZIP6 may be a candidate gene for metastatic spread in estrogen-receptor-positive breast cancer although it remains to be elucidated how ZIP6 mediates the kinetics of zinc and cellular signal pathway. Our findings showed possibility that the suitable species and expression of ZIP are changed by the circumstances around breast cancer cells. Moreover, ZIP10 transcription has been reported to be higher level in the lymph node metastasis-positive group compared with the metastasis-negative group, and to play an essential role in migratory behavior of the invasive and metastatic breast cancer cell line MDA-MB-231 and MDA-MB-435S [28]. Our present data also showed that ZIP10 is required for high glucose induced-migration. This is the first demonstration of the involvement of ZIP10 in estrogen-receptor-positive breast cancer cell, MCF-7, although further studies are needed to clear the functional relation ZIP6 and ZIP10 in migratory activities.

The present study demonstrates that the migratory ability in MCF-7 is promoted in high glucose level, and the Zn^2+^ transported via ZIP6 and ZIP10 is essential function for glucose-induced cell migration. We suggest that ZIP6 and ZIP10 may be candidate markers for metastatic spread in hyperglycemia with diabetes, although molecular mechanisms of studies will be required further, and it is proposed that analyses of high glucose-induced cell migration provide novel strategies for the diagnosis and therapy of breast cancer with hyperglycemia.
